# Pathology of the Prostate Gland

**Published:** 1871-11

**Authors:** 


### Pathology of the Prostate Gland.

Dr. Kraus states it may now be laid down as a rule, admitting of but few exceptions, that all diseases of the prostate take their origin in catarrh of the urethra or bladder. In consequence of the entrance of large quantities of the catarrhal secretion the gland becomes greatly swollen and enlarged, and the entrance of the secretion he attributes to the loss of tone in the bladder, by which the secretion is arrested in the prostatic portion of the urethra, and, subjected to pressure, thus is forced into its ducts. The cavity of the caput gallinaginis also becomes filled with the secretion, and from thence the catarrhal inflammation spreads along the ejaculatory ducts to the vesiculæ and epididymis. In some cases copulative power becomes lost by the agglutination or entire adhesion of the ejaculatory ducts, bloody semen occurs when in hæmorrhagia or vesical catarrh the semen is forcibly expelled through the adherent ducts. Muscular tissue is so prevelent in its texture that the formation of abscess in its substance is a very rare occurrence. Strictures of the urethra from enlargement of the prostate are also of extreme rarity, as the urethra has a large play between the corpora cavernosa, and can exert much locomotion before being interfered with by enlargement of the prostate.—*Med. Times and Gazette.*

